# Empirical Comparison of Tropical Maize Hybrids Selected Through Genomic and Phenotypic Selections

**DOI:** 10.3389/fpls.2019.01502

**Published:** 2019-11-22

**Authors:** Yoseph Beyene, Manje Gowda, Michael Olsen, Kelly R. Robbins, Paulino Pérez-Rodríguez, Gregorio Alvarado, Kate Dreher, Star Yanxin Gao, Stephen Mugo, Boddupalli M. Prasanna, Jose Crossa

**Affiliations:** ^1^ Global Maize Program, International Maize and Wheat Improvement Center (CIMMYT), Nairobi, Kenya; ^2^ School of Integrative Plant Sciences, Cornell University, Ithaca, NY, United States; ^3^ Colegio de Postgraduados, Montecillos, Mexico; ^4^ Genetic Resources Program, International Maize and Wheat Improvement Center (CIMMYT), Texcoco, Mexico

**Keywords:** phenotypic selection, genomic selection, genetic gain, maize, well-watered and water stress environments

## Abstract

Genomic selection predicts the genomic estimated breeding values (GEBVs) of individuals not previously phenotyped. Several studies have investigated the accuracy of genomic predictions in maize but there is little empirical evidence on the practical performance of lines selected based on phenotype in comparison with those selected solely on GEBVs in advanced testcross yield trials. The main objectives of this study were to (1) empirically compare the performance of tropical maize hybrids selected through phenotypic selection (PS) and genomic selection (GS) under well-watered (WW) and managed drought stress (WS) conditions in Kenya, and (2) compare the cost–benefit analysis of GS and PS. For this study, we used two experimental maize data sets (stage I and stage II yield trials). The stage I data set consisted of 1492 doubled haploid (DH) lines genotyped with rAmpSeq SNPs. A subset of these lines (855) representing various DH populations within the stage I cohort was crossed with an individual single-cross tester chosen to complement each population. These testcross hybrids were evaluated in replicated trials under WW and WS conditions for grain yield and other agronomic traits, while the remaining 637 DH lines were predicted using the 855 lines as a training set. The second data set (stage II) consists of 348 DH lines from the first data set. Among these 348 best DH lines, 172 lines selected were solely based on GEBVs, and 176 lines were selected based on phenotypic performance. Each of the 348 DH lines were crossed with three common testers from complementary heterotic groups, and the resulting 1042 testcross hybrids and six commercial checks were evaluated in four to five WW locations and one WS condition in Kenya. For stage I trials, the cross-validated prediction accuracy for grain yield was 0.67 and 0.65 under WW and WS conditions, respectively. We found similar responses to selection using PS and GS for grain yield other agronomic traits under WW and WS conditions. The top 15% of hybrids advanced through GS and PS gave 21%–23% higher grain yield under WW and 51%–52% more grain yield under WS than the mean of the checks. The GS reduced the cost by 32% over the PS with similar selection gains. We concluded that the use of GS for yield under WW and WS conditions in maize can produce selection candidates with similar performance as those generated from conventional PS, but at a lower cost, and therefore, should be incorporated into maize breeding pipelines to increase breeding program efficiency.

## Introduction

With more than 35 million ha harvested each year, maize is the most important staple food crop in sub-Saharan Africa (SSA). In SSA countries, maize is commonly grown by resource-poor farmers and covers large areas with very low average grain yield (1.4 ton/ha) (Smale et al., 2011). The low productivity of maize in SSA is due to several factors including drought and low soil nitrogen stress, foliar diseases, and insect pests among others. The ability to quickly develop germplasm with resistance to important abiotic and biotic stresses will be critical for the resilience of Africa’s maize-based cropping systems in the face of climate change. Breeding for drought tolerance and yield stability is an important objective of maize breeding programs in SSA, and a high priority for the International Maize and Wheat Improvement Center (CIMMYT) (Bänziger et al., 2006; Beyene et al., 2017; Cairns and Prasanna, 2018). Over the past decades, CIMMYT and its partners have made significant progress developing maize germplasm that is tolerant to drought, low soil nitrogen, and diseases including maize lethal necrosis (Beyene et al., 2017; Cairns and Prasanna, 2018). To accelerate breeding for drought tolerance, CIMMYT and its partners adopted several breeding approaches including pedigree selection, marker-assisted recurrent selection (MARS), and genomic selection (GS) combined with high-throughput phenotyping, doubled haploids (DH), and year-round nurseries (Beyene et al., 2016; Cairns and Prasanna, 2018).

Genomic prediction is an approach that uses molecular marker data to predict the genetic value of complex traits in progeny for selection and breeding (Meuwissen et al., 2001). When genomic predictions are used to make selections, the process is referred to as GS. The primary difference between GS and traditional forms of marker-assisted selection (MAS) is the simultaneous use of many markers distributed genome-wide, as opposed to a small set of markers linked to quantitative trait loci (Heffner et al., 2009). The objective of GS is to determine the genetic potential of an individual instead of identifying the specific quantitative trait loci. GS could be used in plant breeding programs in rapid recombination cycles and predict the breeding value of untested parents (genomic estimated breeding value, GEBV). Another way of using GS is with sparse testing where some lines are tested in some environments but predicted in others. Implementing genomic prediction and selection requires development of appropriate training sets consisting of individuals that have been both phenotyped and genotyped, followed by model calibration. Bernardo and Yu (2007) were the first to report the use of GS in maize breeding using simulation data. Massman et al. (2013) used real data to compare GS and MARS in a bi-parental maize population derived from temperate lines and reported that GS gave a 14 to 50% advantage over MARS for grain yield and stover quality. Beyene et al. (2015) reported genetic gains through GS in eight CIMMYT tropical bi-parental maize populations evaluated under managed drought conditions in SSA. The authors showed that (i) the average gain per cycle from GS was 0.086 t/ha under managed drought conditions, (ii) the average grain yield of cycle 3-GS-derived hybrids was significantly higher than that of hybrids derived from C_0_, and (iii) three GS cycles can be achieved in one year. On the other hand, the average gain per cycle using MARS across 10 populations was 0.051 t/ha per cycle under managed drought stress (Beyene et al., 2016). Vivek et al. (2017) reported that the realized genetic gain per year was higher for GS than for phenotypic selection (PS) in two bi-parental populations.

As pointed out by Crossa et al. (2017), the main advantages of GS as compared to phenotype-based selection in breeding are: (i) GS reduces the cost per cycle, and (ii) it increases time efficiency of variety development. For example, in terms of cost reduction in maize breeding, the breeder can testcross 50% of all available lines, evaluate them in first-stage multi-location trials, and then use the phenotypic data to predict the remaining 50% by GS. The time efficiency advantage over PS could come from the second selection cycle, which uses the training population from the previous cycle to predict the new inbred lines, thus excluding testcross formation and first-stage multi-location evaluation trials. As more robust, multi-year training sets are developed, GS can be used to advance the best selection candidates directly to the second stage of multi-location evaluations. This significantly reduces the cost of testcross formation and evaluation in the earliest stage of multi-location multi-year evaluation.

Although testing predictive ability is critical for gathering information for GS, there is a large gap between the findings of these studies and their application in breeding programs (Bernardo, 2016). In maize breeding, the potential of GS was empirically evaluated (Crossa et al., 2010; Windhausen et al., 2012; Massman et al., 2013; Beyene et al., 2015). CIMMYT maize breeding programs have evaluated several GS-related methods with varying levels of success over the past 8 years (Crossa et al., 2010; Burgueño et al., 2012; Windhausen et al., 2012; Beyene et al., 2015; Edriss et al., 2017; Zhang et al., 2017). More recently, some authors have considered using genomic models for predicting hybrid performance (Kadam et al., 2016; Cantelmo et al., 2017; Acosta-Pech et al., 2017; Vélez Torres et al., 2018); these studies have shown that genomic models can give reasonably accurate predictions of the agronomic performance of hybrids. Several of those studies have investigated the accuracy of genomic predictions but there is little empirical evidence on the practical performance of lines selected based on phenotype and GS (untested lines selected solely based on GEBV) in advanced yield trials.

The current study compares the performance of maize DH line testcrosses selected based on GS versus PS in second stage multi-location yield trials of the CIMMYT maize breeding program in SSA. For this study, we used two experimental maize data sets: first-stage multi-location yield trials (hereafter referred to as stage I) and second-stage multi-location yield trials (hereafter referred to as stage II). The stage I data set consisted of 1492 DH lines genotyped with rAmpSeq (epeat lification uencing) dominant sequence tag markers (https://doi.org/10.1101/096628). A subset of these lines (855) was crossed with an individual single-cross tester chosen to complement each specific population and evaluated in replicated trials under optimum and drought conditions for grain yield and other agronomic traits, while the remaining 637 DH lines were predicted using the 855 lines as a training set. The second data set (stage II) consisted of 348 DH lines from the first data set (stage I), of which 172 lines were selected solely based on GEBVs, and 176 lines were selected based on phenotypic performance. In this second data set, each of the 348 DH lines was crossed with three common testers from complementary heterotic groups, and the resulting 1042 testcross hybrids and six commercial checks were evaluated in 4-5 optimum locations and one location with managed drought conditions in Kenya. The objectives of this research were to (1) empirically compare the performance of tropical maize hybrids selected through PS and GS under stress and non-stress conditions, and (2) compare the cost–benefit of genomic and PS in tropical maize.

## Materials and Methods

### Plant Materials Used in the Study

The first data set (stage I) comprised a total of 1492 DH lines derived from 12 bi-parental DH populations developed at CIMMYT’s Maize DH facility in Kiboko, Kenya. The 12 source populations were obtained by crossing elite CIMMYT maize lines (CMLs) with La Posta Seq C7, a drought tolerant population developed at CIMMYT, Mexico, through recurrent selection among full sib/S1 families (Edmeades et al., 1999). The selected CMLs were drought tolerant lines that have good combining abilities and are adapted across several environments in SSA (Beyene et al., 2013). The DH lines were grown at the Kenya Agricultural and Livestock Research Organization Kiboko Research Station during the 2015/16 short rainy season. Based on the results of *per se* evaluation (germination and good stand establishment, plant type, low ear placement, and well-filled ears), 1492 DH lines were selected for stage I multi-location yield trials (**Table 1**). The smallest DH family comprised 34 lines, while the largest had 240 lines.

**Table 1:** List of 12 bi-parental maize populations used in this study.

No.	Population name	# Doubled haploid (DH) lines genotyped	# DH lines phenotyped
1	CML440/LPS-F64	34	34
2	CML445/LPS-F64	181	91
3	CML312/LPS-F64	185	93
4	CML442/LPS-F64	240	126
5	CML505/LPS-F64	162	81
6	CZL04003/LPS-F64	134	67
7	CML536/LPS-F64	180	86
8	CML537/LPS-F64	110	55
9	CML538/LPS-F64	40	40
10	CML540/LPS-F64	51	51
11	ZEWAc1F2-134-4-1-B-1-B*4-1-2-B-B/LPS-F64	75	75
12	CML312/CML540	100	52
	**Total**	**1492**	**851**

### Field Evaluation of Stage I Hybrid Yield Trials

To implement GS in CIMMYT’s maize breeding program, nearly half (855) of 1492 selected DH lines were crossed with a single-cross tester from complementary heterotic group and phenotyped across locations. The 855 hybrids were divided into 14 trials connected by common checks. In each trial, three to six commercial checks were included and planted in an alpha-lattice design with two replications and phenotyped in three well-watered (WW) environments and one managed drought stress (WS) environment in Kenya during the 2017 growing season. The WS experiment was conducted during the dry (rain-free) season by suspending irrigation starting 2 weeks before flowering until harvest, whereas the WW experiments were conducted during the rainy season, applying supplemental irrigation as needed. Entries were planted in two-row plots, 5 m long, with 0.75 m spacing between rows and 0.25 m between hills. Two seeds per hill were initially planted and then thinned down to one plant per hill three weeks after emergence to obtain a final plant population density of 53,333 plants per hectare. Fertilizers were applied at the rate of 60 kg N and 60 kg P_2_O_5_ per ha, as recommended for the area. Nitrogen was applied twice: at planting and 6 weeks after emergence. Fields were kept free of weeds by hand weeding. The following traits were measured: grain yield (GY, tons ha^− 1^), anthesis date (AD, days), plant height (PH, cm), grain moisture (MOI, %), gray leaf spot (GLS, 1–5 rating score), and turcicum leaf blight (TLB, 1–5 rating score). Plots were manually harvested and GY was corrected to 12.5% moisture. AD was measured from planting to when 50% of the plants shed pollen, and PH was measured from the soil surface to the flag leaf collar on five representative plants within each plot.

### Genotyping 1492 DH Lines Using rAmpSeq

Leaf samples were taken from each of the 1492 DH lines and sent to Intertek, Sweden, for DNA extraction. The DNA sample plates were forwarded to the Institute for Genomic Diversity, Cornell University, Ithaca, NY, USA, for genotyping with repetitive sequences (rAmpSeq markers) as per the procedure described by Buckler et al. (2016). Each sample was first amplified with PCR, and DNAs within each batch were pooled and multiplexed for rAmpSeq sequencing, a new genotyping technology which is used to amplify repetitive (LTR/retroelements) regions of the genome. A K-mer based approach was used to design the primer pairs which range between 150-bp to 200-bp in length and target ∼1,500–2,000 loci in the genome (Buckler et al., 2016, http://www.biorxiv.org/content/early/2016/12/24/096628). With the availability of thousands of adaptors, this technology makes it possible to genotype 3,000 samples in a single sequencing run and dramatically reduces the genotyping cost per sample. A total of 4657 markers that passed quality control were used for GS.

### Field Evaluation of Advanced (Stage II) Hybrid Trials Selected Through Phenotypic and GS

From stage I analyses, the top performing 348 (23%) DH lines were chosen for stage II evaluation. Among these 348 DH lines, 172 lines represented selection from the 637 genomic predicted lines that had above average GEBVs and 176 lines were selected from the 855 phenotyped lines that had above average Best Linear Unbiased Estimates (BLUE). Each of these DH lines were crossed with three common testers from complementary heterotic groups. The resultant 1042 testcross hybrids were evaluated in eight connected trials. Six commercial checks were included in each trial and planted in an alpha-lattice design with two replications and phenotyped in 4-5 WW environments and one WS environment in Kenya in 2018. The WS experiment was conducted during the dry (rain-free) season by suspending irrigation starting 2 weeks before flowering until harvest, whereas the WW experiments were conducted during the rainy season, applying supplemental irrigation as needed. Planting and agronomic managements were similar as explained for stage I trials. The following traits were measured: grain yield (GY, tons ha^−1^), anthesis date (AD, days), plant height (PH, cm), grain moisture (MOI, %), gray leaf spot (GLS, 1–5 rating score), and turcicum leaf blight (TLB, 1–5 rating score). Plots were manually harvested and GY was corrected to 12.5% moisture.

### Phenotypic Data Analysis

There were two sets of phenotypic field trials; the first set included 855 hybrids used to predict the performance of unobserved 637 lines (stage I), and the second set (stage II) was made up of 1042 hybrids from 348 DH lines (172 lines selected from GEBV alone and 176 lines selected based on phenotypic data) crossed with three testers. Note that the second set of field trials was used to compare the performance of the GS vs PS of hybrids. All the phenotypic analyses were done to obtain the variance components and BLUEs for the lines under WW and WS. All testcrosses were evaluated in different trials but adjacent to each other and connected by common checks in the same field. Phenotypic data was analyzed first within trials and then across trials.

The BLUEs across WW and WS locations for each trial and each trait were generated using the following linear mixed model carried out using the META-R software (Alvarado et al., 2017):

$$Y_{ijrk}\;=\;\mu \;+\;L_{j}+\;R_{r}\left(L_{j}\right)+\;B_{k}\left[R_{r}\left(L_{j}\right)\right]+\;G_{i}+\;GL_{ij}+\;\epsilon_{ijrk}$$

where *Y* *_ijrk_* is the grain yield of genotype *i* at location *j* in replicate *r* within block *k*; µ is the general mean; *L* *_j_* is the fixed effect of location *j*; *R* *_r_* *(L* *_j_* *)* is the fixed effect of replicate *r* within location *j*; *B* *_k_*[*R* *_r_*(*L* *_j_*)] is the random effect of incomplete block *k* within replicate *r* and location *j* is assumed to be independently and identically normal distributed with mean zero and variance $\sigma_{\;B(RL)}^{2}$;*G* *_i_* is the effect of line *i*; *GL* *_ij_* is the effect of the line × location interaction; and $\epsilon_{ijrk}$ is the random residual error assumed independent and identically normal distributed with mean zero and variance $\sigma_{\;\;\epsilon }^{2}$. The variance components and heritability across locations for WW and WS sites were computed. The BLUE of each trait for the single WS location are obtained from the following model

$$Y_{irk}\;=\;\mu \;+\;R_{r}+\;B_{k}(R_{r})+\;G_{i}+\;\epsilon_{irk}$$

The analysis across trials was also performed using similar model as those shown above but including the trial as fixed effect.

### Genomic-Enabled Prediction Models for Stage-I Yield Trial Data

The BLUE of the entries within and across testers were used for genome-based predictions. GEBVs were calculated for GY, AD, MOI and PH using the BGLR statistical R-package (Pérez-Rodríguez and de los Campos, 2014) within and across testers for WW and WS sites. For genome-enabled prediction, a total of 4657 markers that passed quality control were selected. For GS, the Genomic Best Linear Unbiased Predictor (G-BLUP) model was employed. Further, to understand the effect of testers on prediction accuracy, GS was applied to predict unobserved lines within and across testers.

The models described below were used with two purposes: one was to use the 855 lines as a training set to predict the GEBV of 637 lines (testing set) and use the observed and predicted values to select top performing lines. The other objective of the models described below was to study the genome-based prediction accuracy of the 855 lines with phenotypic and genotypic data and determine the prediction accuracy using main effects and main effects plus interaction models for each tester and across testers.

### Environment + Genome Model (E + G + e)

This model can be expressed as

$$y_{ij}=\mu +E_{i}+g_{j}+e_{ij},$$

Where *y* *_ij_* is the response trait for the *j* ^th^ hybrid in the *i* ^th^ environment, *µ* is the overall mean, and *E* *_i_* is the fixed effect of the site (either WW or WS). Here, *g* *_j_* corresponds to the genomic breeding value of the *j* ^th^ line defined as a linear combination of marker codes and the corresponding marker effects, such that $g_{j}=\sum_{m=1}^{p}x_{jm}b_{m},$ where *p* is the number of markers, *x* *_jm_* is the marker code for the *j* ^th^ line at the *m* ^th^ marker position (*m* = 1,…, *p*), and *b* *_m_* is the corresponding marker effect. The marker effects are assumed identically and independently distributed (IID) such that $b_{m}\overset{\text{IID}}{\sim }N\left(0,\sigma_{b}^{2}\right),$ with $\sigma_{b}^{2}$ being the variance component of the marker effects. The covariance matrix of the vector of genomic values *g* = {*g* *_j_*} can be written as $\mathrm{Cov}\left(g\right)=G\sigma_{g}^{2}$ where *G* is the genomic relationship matrix computed as $G=\frac{XX^{'}}{p}$ where *X* is the standardized genotype matrix (by columns), *p* is the number of markers, and $\sigma_{g}^{2}=p\times \sigma_{b}^{2}$ denotes the genomic variance component. Hence, $g=\left\{g_{j}\right\}\sim N\left(0,G\sigma_{g}^{2}\right)$ Since molecular marker information varies across individuals (even within the same family), the estimated breeding values are unique for each genotype. Finally, *e* *_ij_* is the residual assumed to have an identical and independent distribution (IID) such that $e_{ij}\overset{\mathrm{IID}}{\sim }N\left(0,\sigma_{e}^{2}\right)$ where $\sigma_{e}^{2}$ is the residual variance.

Note that this model was used for the genomic prediction computed for the WW sites. The predictions for the unique managed sites had only the G + e component because these trials were established in only one managed drought site.

### Environment + Genome + Genome × Environment Model (E +G + GE + e)

This is the same as the previous model but includes the interaction term based on marker and environment interaction data. The model (Jarquín et al., 2014) is written as:

$$y_{ij}=\mu +E_{i}+g_{j}+gE_{ij}+e_{ij},$$

Where *gE* *_ij_* is the component representing the interactions between molecular markers of the *j* ^th^ line and the *i* ^th^ environment. The distributional assumption for this term is such that $gE=\left\{gE_{ij}\right\}\sim N\left(0,\left(Z_{g}GZ_{g}^{'}\right){}^{\circ}\left(Z_{E}Z_{E}^{'}\right)\sigma_{gE}^{2}\right)$ and $\sigma_{gE}^{2}$ is the variance component of the random interaction component *gE*.The other terms were already defined in the previously defined E + G + e model. As already mentioned, this model was used for the genomic prediction computed for the WW sites. The prediction for the unique managed sites includes only the G + e component.

### Random Cross-Validation for Determining the Prediction Accuracy of the Models

The performance of the models when predicting the five traits was evaluated using the average Pearson’s correlation coefficient between observed and predicted values. The random cross-validation scheme mimics real plant breeding situations and is a scheme where the performance of 20% of the maize testcrosses was not observed in any of the environments and the rest of the lines (80%) were already observed in the same target environments. For this scheme, a five-fold random partitioning (80% of the data used as the training set, and the remaining 20% as the testing set) was employed. Four folds were used for training the models and for predicting the remaining fold. This procedure was repeated over the five folds and the predictions from the testing fold were joined in a single vector. Then, Pearson’s correlations between predicted and observed values within the same environment were computed. The partitioning was repeated 100 times. The cost benefits of PS vs GS were analyzed using spreadsheet-based budgeting tools.

## Results

### Test Cross Hybrid Performance in Stage I and II Trials

For stage I, mean GY averaged across WW locations ranged from 3.49 to 9.14 t/ha with an overall mean of 6.03 t/ha, whereas at stage II it improved further, ranging from 5.1 to 11.6 t/ha with an average of 7.59 t/ha (**Table 2**). Under WS, GY ranged from 1.08 to 5.76 t/ha with an overall mean of 3.25 t/ha at stage I, whereas at stage II, the range varied from 0.77 to 6.33 t/ha with an average of 3.23 t/ha. The average GY, PH, AD, and MOI were higher under WW conditions than under WS in stage I trials (**Table 2**). Interestingly, the average performance of hybrids at stage II was also higher for GY, AD, PH, and MOI compared to stage I trials. The magnitude of genotypic variances was higher than the genotype by environment interaction variances for all traits at stage I and only for GY and AD at stage II under WW conditions. For stage I trials, the heritability under WW conditions varied from 0.31 to 0.77, while under WS, it ranged from 0.84 to 0.95. Whereas at Stage II, the heritability under WW conditions varied from 0.18 to 0.82, while under WS it ranged from 0.32 to 0.61. A total 176 lines that had above average phenotypic value for grain yield and other agronomic traits were advanced to stage II evaluation (**Supplementary Table S1**). These superior lines were derived from all 12 populations, suggesting that the donor parents used to develop the DH lines are excellent sources of germplasm for combining ability with good adaptation to eastern Africa.

**Table 2:** Mean, range, genetic variance, and broad-sense heritability estimates for grain yield (GY, t/ha) anthesis date (AD, days), plant height (PH, cm), moisture (MOI, %), gray leaf spot (GLS, 1–5 rating score), and turcicum leaf blight (TLB, 1–5 rating score) for stage I and stage II testcrosses evaluated under optimum and managed drought stress conditions in Kenya.

	Optimum (Stage I)						Managed drought (Stage I)			
	GY	AD	PH	MOI	GLS	TLB	GY	AD	PH	MOI
Mean	6.03	64.31	235.56	16.81	2.31	3.43	3.25	63.3	207.7	16.2
Min	3.49	53.71	194.33	14.13	0.81	1.97	1.08	57.98	163.53	8.05
Max	9.14	73.90	270.04	21.60	4.18	5.04	5.76	69.88	244.65	23.20
Checks Mean	6.14	64.89	246.25	16.08	2.40	2.90	2.99	63.90	222.69	15.96
σ^2^_G_	0.19**	1.29**	41.69**	0.18**	0.01*	0.10**	0.17**	1.96**	49.82**	0.91**
σ^2^_T_	0.00	0.90	11.59	0.36	0.02	0.07	0.52	0.74	0.63	0.43
σ^2^_E_	0.42	145.11	1200.50	14.70	0.00	0.00	–	–	–	–
σ^2^_GxE_	0.18**	0.14**	0.06*	0.00	0.01*	0.10**	–	–	–	–
σ^2^_GxT_	0.74**	3.00**	81.18**	0.81**	0.02**	0.07**	–	–	–	–
σ^2^_e_	1.41	2.82	160.47	3.19	0.16	0.24	0.33	1.36	59.08	2.43
*h* *^2^*	0.46	0.77	0.67	0.31	0.37	0.65	0.88	0.95	0.92	0.84
LSD	2.09	4.11	21.15	2.52	0.89	1.00	1.66	2.50	24.30	3.40
CV	19.66	2.61	5.38	10.62	17.31	14.12	17.56	1.80	3.70	9.70
	Optimum (Stage II)						Managed drought (Stage II)			
Mean	7.59	71.9	247.6	19.64	1.98	2.58	3.23	72.40	206.40	14.60
Min	5.10	62.0	196.7	16.54	1.43	1.50	0.77	63.50	159.60	9.70
Max	11.67	77.5	291.2	22.22	4.45	4.08	6.33	81.00	247.10	22.90
Checks Mean	6.90	69.43	262.94	18.83	2.03	1.76	2.31	71.80	217.50	14.40
σ^2^_G_	0.33**	2.6**	101.0**	0.24**	0.00	0.09**	0.20**	2.30**	73.50**	1.00**
σ^2^_T_	0.37	1.9	97.1	0.00	0.00	0.08	0.55	7.1	44.10	4.60
σ^2^_E_	2.06	75.8	389.5	10.03	0.00	0.01	–	–	–	–
σ^2^_GxE_	0.25**	1.10**	189.3**	2.31**	0.04	0.00	–	–	–	–
σ^2^_GxT_	0.23**	0.20*	10.9**	0.21**	0.01	0.00	–	–	–	–
σ^2^_e_	1.27	2.10	102.3	4.38	0.10	0.19	0.40	1.40	68.90	2.10
*h* *^2^*	0.38	0.14	0.53	0.09	0.82	0.18	0.34	0.32	0.61	0.52
LSD	2.11	2.00	29.30	4.48	0.63	0.66	1.42	2.70	21.10	3.00
CV	14.82	2.00	4.10	10.66	16.20	16.83	19.93	1.70	4.00	9.9

*,** Significance at P < 0.05 and 0.01 level, respectively.

### Genomic-Enabled Predictions of GY and Other Agronomic Traits At Stage I

The cross-validation analyses yielded moderately high prediction correlations among optimum and drought conditions for GY and other agronomic traits. The prediction correlations ranged from 0.65–0.67 for GY, 0.57–0.65 for MOI, 0.67–0.75 for AD, and 0.70–0.72 for PH (**Table 3**). In general, trait–tester combination with higher training set (CML395/CML444) had a higher predication accuracy than the other two testers (CML312/CML395 and CML312/CML442) that had lower training set (**Table 3**). The predication accuracy was lowest for testers CML312/CML442 and CML312/CML395 for all traits and highest for tester CML395/CML444 both under WW and WS conditions.

**Table 3:** Prediction accuracy for each tester and across testers under cross-validation scenarios for grain yield (GY), anthesis date (AD), plant height (PH), and moisture content (MOI) evaluated under well-watered (WW) and water stress (WS) conditions in Kenya.

Trait	Model\Tester	Within tester			Across testers
		CML312 × CML395	CML312 × CML442	CML395 × CML444	
Total Hybrids		111	742	979	
GY-WW	G	0.41 ± 0.09	0.16 ± 0.12	0.60 ± 0.03	0.67 ± 0.05
	G + GE	0.42 ± 0.07	0.19 ± 0.07	0.59 ± 0.04	–
GY-WS	G	0.75 ± 0.04	0.22 ± 0.18	0.64 ± 0.07	0.65 ± 0.05
MOI-WW	G	0.58 ± 0.05	0.16 ± 0.14	0.58 ± 0.01	0.65 ± 0.04
	G + GE	0.61 ± 0.07	0.09 ± 0.07	0.59 ± 0.04	–
MOI- WS	G	0.09 ± 0.04	0.44 ± 0.16	0.61 ± 0.06	0.57 ± 0.05
AD-WW	G	0.58 ± 0.07	0.41 ± 0.13	0.70 ± 0.07	0.75 ± 0.04
	G + GE	0.53 ± 0.19	0.40 ± 0.14	0.74 ± 0.04	–
AD- WS	G	0.51 ± 0.06	0.49 ± 0.20	0.63 ± 0.04	0.67 ± 0.05
PH-WW	G	0.28 ± 0.10	0.14 ± 0.12	0.65 ± 0.03	0.70 ± 0.03
	G + GE	0.40 ± 0.06	0.16 ± 0.09	0.67 ± 0.03	–
PH-WS	G	0.52 ± 0.03	0.17 ± 0.12	0.72 ± 0.04	0.72 ± 0.04

A total 172 lines that had above average GEBVs were advanced to stage II evaluations (**Supplementary Table S1**).

### Comparing Hybrids Developed Through PS and GS Under WW Conditions At Stage II

At stage II, 1042 hybrids were evaluated among them 526 were developed from lines selected based on PS and the remaining were derived from lines selected based on GEBVs. The GY of 526 testcross hybrids advanced through PS evaluated across five WW locations (hereafter referred to as PS-WW) ranged from 5.54 to 11.67 t /ha (**Supplementary Table S2**). In the PS-WW, the top 15% of hybrids produced an average grain yield of 9.4 t ha^−1^, which represents an increase of 1.0 t ha^−1^ compared to the best commercial check, which produced 8.4 t ha^−1^. The best hybrid advanced through PS yielded 39% and 63% more than the best commercial check and the mean of commercial checks, respectively (**Table 4**).

**Table 4:** Comparison of hybrids advanced through genomic and phenotypic selections and commercial checks evaluated at stage II or advanced yield trials under optimum and managed drought stress across Kenya in 2018.

	Phenotypic selection (PS)		Genomic selection (GS)	
	Well-watered (GY, t/ha)	Water stress (GY, t/ha)	Well-watered (GY, t/ha)	Water stress (GY, t/ha)
All hybrids	7.7	3.2	7.5	3.2
Top 15% of hybrids	9.4	4.7	9.1	4.8
Best hybrid	11.7	6.2	10.4	6.3
Mean of commercial checks	7.2	2.3	7.2	2.3
Best check	8.4	3.3	8.4	3.3
**Yield Improvement**				
Top 15% of hybrids over commercial checks	23%	51%	21%	52%
Top 15% of hybrids over the best commercial check	12%	42%	8%	47%
The best hybrid over commercial checks	63%	169%	46%	175%
The best hybrid over the best commercial check	39%	88%	24%	92%

A total of 516 hybrids advanced through GS and evaluated at the same five WW locations (hereafter referred to as GS-WW) produced GY ranging from 5.1 to 10.44 t/ha (**Supplementary Table S2**). The top 15% of hybrids advanced through GS had a mean GY of 9.1 t/ha, which represents a 0.7 t/ha increase over the yield of the best commercial check. The top 15% of hybrids had an average yield advantage of 8 and 21% over the best check and the mean of the checks, respectively (**Table 4**). The best hybrid advanced through GS had 24 and 46% higher GY than the best commercial check and the mean of the commercial checks, respectively.

The top 15% of hybrids advanced through PS on average had an increase of 8.5 cm in PH and 4.7 days in AD and a 1% increase in grain moisture content compared to the mean of the commercial checks (**Supplementary Table S2**). However, there was no difference between the top 15% of hybrids and the commercial checks in their responses to the two main foliar diseases, GLS and TLB (**Supplementary Table S2**). The top 15% of hybrids advanced through GS had a 6 cm increase in PH and 4.7 days in flowering and a 1% increase in grain moisture content compared to the mean of the commercial checks (**Supplementary Table S2**). There was no difference among the top 15% of hybrids advanced through PS and GS for AD, MOI and their responses to the two main foliar diseases, GLS and TLB (**Supplementary Table S2**).

### Comparing Hybrids Developed Through PS and GS Under Managed Drought Stress

A total of 526 hybrids advanced through PS were also evaluated under managed drought stress (hereafter referred to as PS-WS); their mean GY ranged from 0.99 to 6.19 t ha^−1^ (**Supplementary Table S3**). The top 15% of hybrids (79 hybrids) produced 42% and 51% higher mean GY than the mean GY of the best commercial check and the mean of the checks, respectively (**Table 4**). The best hybrid advanced through PS produced 88% and 169% higher GY than the best check and the mean of the commercial checks, respectively, while for 516 hybrids that were advanced through GS, their GY performance under WS ranged from 1.14 to 6.33 t/ ha (**Supplementary Table S3**). The top 15% of hybrids produced 47% and 52% higher mean GY than the best commercial check and the mean of checks, respectively (**Table 4**).

The best hybrid advanced through GS produced 92% and 175% higher GY than the best check and mean of commercial checks, respectively (**Table 4**). Compared to the mean of commercial checks, the top 15% of hybrids advanced through PS and GS had a 3% increase in MOI, 2.8 days in AD, and a 10 cm increase in PH (**Supplementary Table S3**).

### Advancement Rate of Stage II Candidates to Stage III

**Figure 1:**
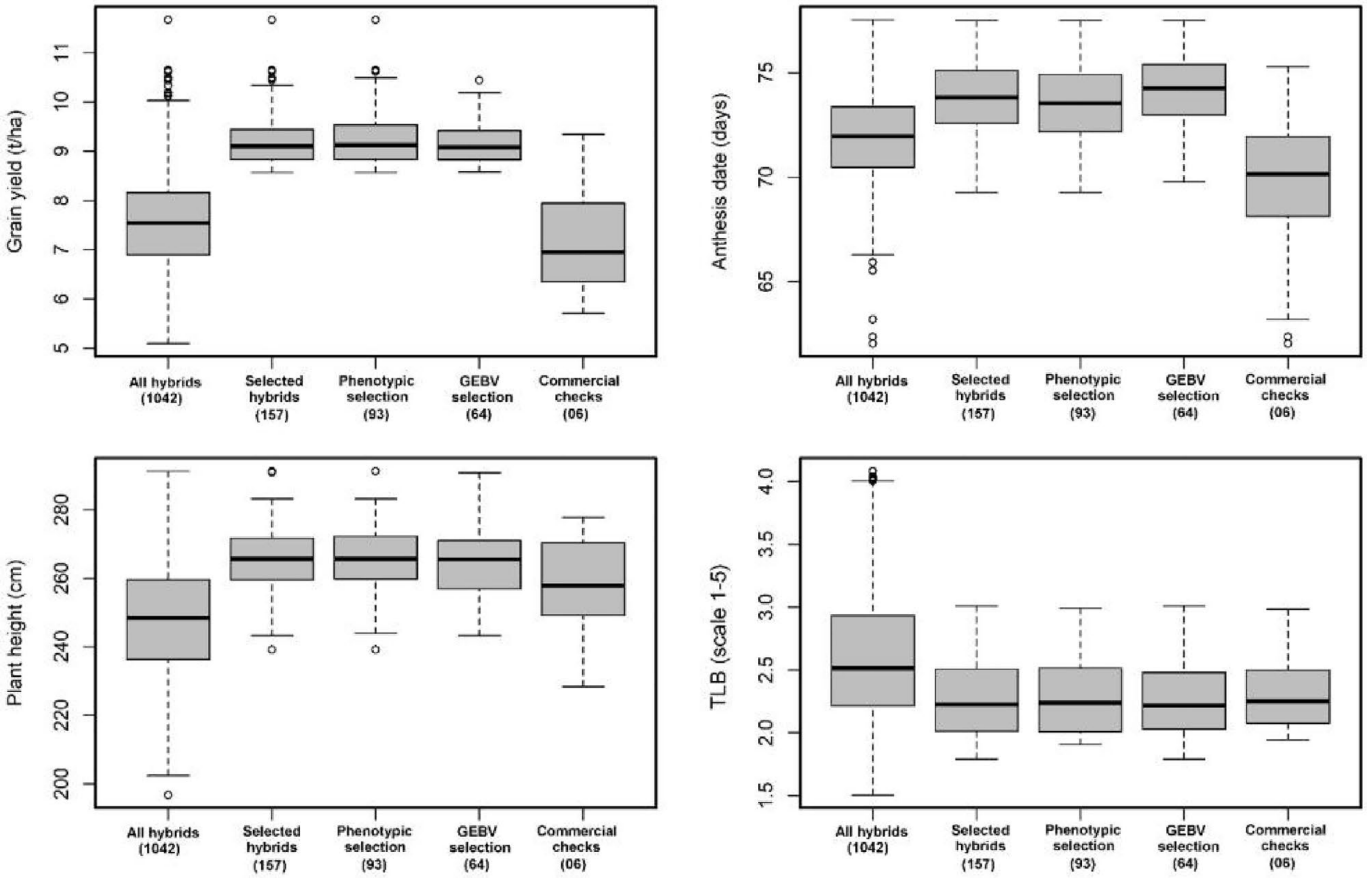
Performance of hybrids advanced through genomic selection (GS), phenotypic selection (PS), and commercial checks evaluated in stage II trials under optimum conditions for grain yield (GY, t/ha), anthesis date (AD, days), plant height (PH, cm), and turcicum leaf blight (TLB, 1–5 rating score). The numbers in the bracket indicated the total number of hybrids.

**Figure 2:**
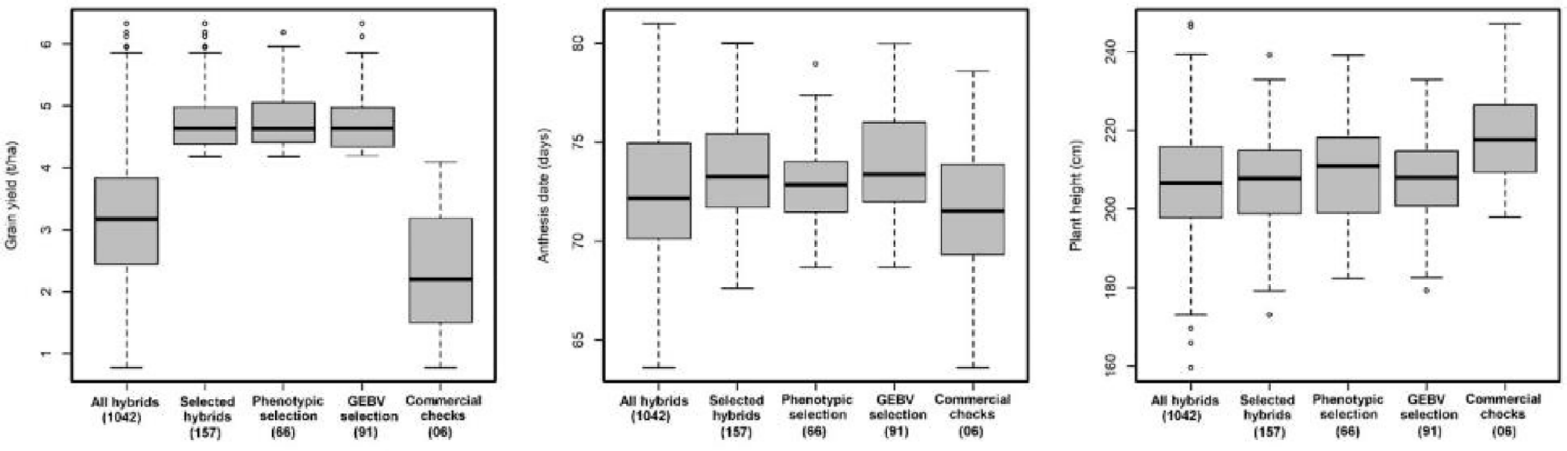
Performance of hybrids advanced through GS and PS and commercial checks evaluated in stage II trials under managed drought for GY (t/ha), AD (days), and PH (cm). The numbers in the bracket indicated the total number of hybrids.

An additional metric of interest when considering the overall efficacy of GS as a substitute for conventional PS only schemes is the advancement rate of the GS stage II cohort compared with the advancement rate of the PS stage II cohort. The actual advancement rate of the two methods is a useful means of comparing the overall value of the two groups of advanced lines since it captures all information that the breeder uses to make the decision whether or not to move a given DH line/s into advanced testing (**Figures 1** and **2**). Under WW condition the top 15% (157 of 1042 hybrids) advanced to stage III trials, 93 hybrids were developed through PS, and 64 hybrids were advanced thought GS (**Figure 1**).

While under WS condition the top 15% (157 of 1042 hybrids) advanced to stage III trials, 91 hybrids were developed through GS, and 66 hybrids were advanced through PS. There was no significant difference among the top 15% of hybrids advanced through PS and GS for grain yield, AD, and PH (**Figure 2**). The overall advancement rate of GS stage II candidates was 41% and 58% compared with an advancement rate of 59% and 42% for PS stage II candidates under WW and WS condition, respectively, indicating that the two groups of selection candidates had functionally equivalent potential in terms of producing new stage III candidates.

### Cost–Benefit Analysis, PS Versus GS

We compared the costs involved in PS and GS using spreadsheet-based budgeting tools (**Table 5**). At present, for a single entry to make testcross and conduct two row plot yield trial costs US$ 15 in Kenya. This value represented lower boundary cost because it was mainly based on operational costs excluding personnel and other costs. The cost of genotyping an entry is US$ 10. Based on this rough estimate, developing and evaluating 1492 testcrosses in stage I trials at four locations with two replications per location would have cost US$ 134,480, while it costs US$ 91,870 using a combination of phenotypic and GS. Therefore, using the current method (phenotyping half of the materials and predicting the remaining half), the same outcome was achieved with 68% of the phenotyping costs (**Table 5**). These costs are likely to vary in different breeding programs (primarily due to differences in labor costs), but the results indicated that GS was relatively cheaper than PS.

**Table 5:** Cost–benefit analysis of phenotypic selection and genomic selection in International Maize and Wheat Improvement Center’s (CIMMYT’s) maize breeding program in Kenya.

Methods	Cost/entry (US$)	No. of entries	No. of reps/sites	No. of rows/sites	No. of sites	Total cost (US$)
PS (making testcrosses)	10	1492	1	1	1	14,920
PS (stage I multi-location yield trials)	5	1492	2	2	4	119,360
GS (making testcrosses)	10	855	1	1	1	8,550
GS (phenotyping training set in stage I multi-location yield trials)	5	855	2	2	4	68,400
GS (genotyping all lines)	10	1492	1	1	1	14,920
Total cost of GS						91,870
Total cost of PS						134,280
GS:PS cost ratio						0.68

## Discussion

With the advent of DH technology, thousands of fixed lines are generated each year in maize. However, identifying the best genotypes requires extensive field evaluations with several hybrid combinations, and all DH lines cannot be evaluated because of limited space and resources. One method for reducing the number of hybrids for field evaluation is crossing all DH lines with a common tester in the early stages of a breeding cycle. Another method is to use a genetic similarity matrix derived from pedigree or molecular markers for predicting performance of untested crosses (Kadam et al., 2016; Cantelmo et al., 2017). GS can be used in plant breeding to improve selection in the early stages of a breeding program without testing all available lines in yield trials. Recently several genomic prediction models (reviewed by Crossa et al., 2017) were used to predict the performance of tested and untested hybrids, and those predictions can be used to decide which hybrids should be selected for further evaluation in field trials.

In this study, we compared the performance of maize DH lines selected from stage I multi-location yield trials based on BLUEs and GEBVs by evaluating the hybrids in common stage II multi-location yield trials of the CIMMYT maize breeding program. We evaluated a total of 855 hybrids under optimum and drought conditions and used BLUEs data to predict the remaining 637 hybrids which were genotyped but have never been phenotyped. In our study, the prediction accuracy for GY under WW conditions was 0.67, and under WS, it was 0.65 (**Table 3**). Our results agree with previous maize studies by Crossa et al. (2010) and Zhang et al. (2017), who reported medium to high prediction accuracy. In our study, higher marker density, higher heritabilities, and similarity in training and prediction data sets gave higher accuracies of estimated breeding values. Lorenz and Smith (2015) reported that prediction accuracies for GS decreased as the similarity of individuals in the training and test populations decreased. In our case, the training populations used for GS are highly related and purposefully designed (genotyping all and phenotyping half) for reducing field phenotyping, which is costly and logistically complex. Our results agree with those of previous studies by Riedelsheimer et al. (2012) and Lariepe et al. (2017), who concluded that prediction accuracies are enough to make GS more efficient than PS.

Identification of optimum size as training and prediction set is crucial for implementing GS in maize breeding program. Cao et al. (2017) reported that high predication accuracy was observed when 50% of the total genotypes were used as a training population. Zhang et al. (2019) obtained moderate-to high predication accuracy trait–environment combinations, when half of the population is used to build the prediction model. In this study we have implemented genotyping all and phenotyping half prediction scheme to reduce the cost of phenotyping all DH line generated each year in stage I yield trials. The ultimate objective is to select untested lines based on GEBV from previous years that improve accuracy and go directly to Stage II yield trials by skipping stage I yield trials. This will require to build multi-year estimation set for specific germplasm groups and targeted growing regions.

### Comparing Performance of Hybrids Advanced to Stage II Under WW Conditions

The mean GY of hybrids advanced through GS and PS methods was significantly higher than the mean of the commercial checks (**Figure 1**). The top 15% of hybrids advanced through PS were slightly better than hybrids advanced through GS. The top 79 hybrids (15% SI) advanced through PS had mean GY of 9.4 t/ha, while the top 15% of hybrids (77 hybrids) advanced through GS had mean GY of 9.1 t/ha under WW conditions. When historical data from the same breeding program is available, there is the potential to bypass stage I trial evaluation and move material directly into stage II. This approach would reduce both the costs and cycle time but will require accurate predictions from training sets composed of historical data. GS has the potential for increasing the genetic gain per year by accelerating the breeding cycles (Crossa et al., 2017). Beyene et al. (2015) reported that GS can save the time over PS because three rapid cycles of recombination were possible to complete in a year. Gaynor et al. (2016) using simulation data proposed a two-part strategy for GS in plant breeding: namely, population improvement and product development. The population improvement strategy uses GS to perform rapid cycles of recurrent selection to minimize breeding cycle time and maximize the genetic gain per year, while the product development component focuses on developing inbred lines as hybrid parents. The authors concluded that implementing GS in breeding programs increases breeding program efficiency by reducing the cost of achieving a similar outcome.

### Comparing Performance of Hybrids Advanced to Stage II Under WS Conditions

Comparison of hybrids advanced through PS and GS under drought stress conditions revealed that GS did slightly better (4.68 t/ha was the mean of the top 15% of hybrids) than PS (4.48 t/ha, mean of the top 15% of hybrids). There was no significant difference among the top 15% of hybrids advanced through PS and GS for other traits. Longin et al. (2015) found an increased genetic gain when selecting parents based entirely on GEBV for highly heritable traits in wheat. The ultimate objective is to select untested lines based on GEBV from previous years that improve accuracy and go directly to advanced yield trials by skipping preliminary stage I yield trials. In CIMMYT maize breeding program, GS could be implemented to predict untested lines at the early stage of testing by selecting DH with good GEBV and going directly to stage II trials. This could reduce each breeding cycle to less than 2 years. The greatest benefit of GS for achieving genetic gains in crops will come from decreased cycle time (Heffner et al., 2009), as has been predicted and observed in GS of dairy cattle (Schaeffer, 2006; García-Ruiz et al., 2016).

### Cost–Benefit Analysis of GS vs. PS

GS was found to outperform MAS using the same financial investment, even at low prediction accuracies (Bernardo and Yu, 2007; Heffner et al., 2010). GS can be used to identify promising lines much sooner than PS, thereby reducing cycle time and increasing the genetic gain per year (Heffner et al., 2009). Several studies considered the economic aspects of plant breeding while comparing the evaluation of selection strategies. Abalo et al. (2009) found that compared to PS, MAS had 26% lower total operating costs for maize streak virus resistance. For GS, Heffner et al. (2010) found similar results in maize and winter wheat (*Triticum aestivum* L.). Comparison of the genetic gain per unit time and per unit cost for oil palm (*Elaeis guineensis* Jacq.) breeding under PS and GS also revealed higher genetic gain per unit cost for GS (Wong and Bernardo, 2008). In our study, GS reduced the cost by 32% over PS with similar selection gains. Currently, we are testing another set of lines with the aim of using historical data to predict new lines and bypass the first stage of testing, and also using both pedigree and marker data to improve the prediction accuracy, which significantly reduces costs and shortens the breeding cycle.

## Conclusions

The largest potential advantage of GS is predicting the breeding value of genotyped parents that were never phenotyped. We found similar responses to selection using PS and GS for grain yield under WW and WS conditions. The top 15% of hybrids advanced through GS and PS produced 21% to 23% higher GY under WW and 52% to 51% under WS than the mean of the commercial checks. The GS reduced the cost by 32% over the PS with similar selection gains. We conclude that the use of GS for yield under optimum and drought conditions in tropical maize can produce selection candidates with similar performance as those generated from conventional PS, but at a lower cost; therefore, this strategy should be effectively incorporated into maize breeding pipelines to enhance breeding program efficiency.

## Data Availability Statement

All datasets generated for this study are included in the article/**Supplementary Material**.

## Author Contributions

YB, MG, MO, BP, JC, KR, and SM contributed in the project planning and overall coordination. YB performed and coordinated the field experiments. MG, SG, and KD were responsible for coordinating sample management and genotyping. JC, PP, KR, GA, MG, and YB carried out the analysis. YB, JC, and MG wrote the manuscript. All authors have made their contribution in editing the manuscript and approved the final version.

## Conflict of Interest

The authors declare that the research was conducted in the absence of any commercial or financial relationships that could be construed as a potential conflict of interest.
